# Quality of life associated with nursing professionals’ individual resources and work

**DOI:** 10.1590/0034-7167-2023-0476

**Published:** 2024-06-17

**Authors:** Isabela Saura Sartoreto Mallagoli, Evandro Piccinelli da Silva, Maria Aline do Nascimento Oliveira, Italo Everton Bezerra Barbosa, Aristeia Nunes Sampaio, Aline Bicalho Matias, Dulce Aparecida Barbosa, Angélica Gonçalves Silva Belasco

**Affiliations:** I Universidade Federal de São Paulo. São Paulo, São Paulo, Brazil; II Universidade Federal do Acre. Rio Branco, Acre, Brazil

**Keywords:** Quality of Life, Nursing, Occupational Health, Mental Health, Nurse Practitioners, Calidad de Vida, Enfermería, Salud Laboral, Salud Mental, Enfermeras Practicantes

## Abstract

**Objectives:**

to assess the physical and mental components of nursing professionals’ quality of life and associate them with individual, health and work characteristics.

**Methods:**

cross-sectional research, with nursing professionals from a university hospital in São Paulo. Own questionnaire and validated instruments were applied.

**Results:**

the overall quality of life was compromised. The physical component was lower in relation to low family income and among those who perceived greater control/pressure at work, and better for those who practiced physical activity and had support of leader and organization. The mental component was lower in professionals who reported dissatisfaction with work, worse self-rated physical health and were older. Scores for both components reduced due to work-related illnesses, worse work ability and increased daytime sleepiness.

**Conclusions:**

quality of life was statistically associated with controllable institutional factors and individual resources that, except age, can be promoted.

## RESUMEN

**Objetivos:**

evaluar los componentes físicos y mentales de la calidad de vida de los profesionales de enfermería y asociarlos con características individuales, de salud y laborales.

**Métodos:**

investigación transversal, con profesionales de enfermería de un hospital universitario de São Paulo. Se aplicó cuestionario propio e instrumentos validados.

**Resultados:**

la calidad de vida general se vio comprometida. El componente físico fue menor en relación con bajos ingresos familiares y entre quienes percibieron mayor control/presión en el trabajo, y mejor para quienes practicaban actividad física y contaban con apoyo de la dirección y organización. El componente mental fue menor en los profesionales que refirieron insatisfacción con el trabajo, peor salud física autovalorada y mayor edad. Las puntuaciones de ambos componentes se redujeron debido a enfermedades relacionadas con el trabajo, peor capacidad laboral y aumento de la somnolencia diurna.

**Conclusiones:**

la calidad de vida se asoció estadísticamente con factores institucionales controlables y recursos individuales que, excepto la edad, pueden ser promovidos.

## INTRODUCTION

Nursing is a fundamental element throughout the healthcare system, representing around 59% of the world’s healthcare workforce^(1)^. In Brazil, data from the Federal Nursing Council considers the existence of more than 2.8 million of these professionals, of which 25.66% in the state of São Paulo alone^(2)^.

Nursing professionals’ daily contact with situations of varying levels of complexity, multiple conditions of lack of protection and health risks favors the development of physical and mental dysfunctions^(3,4)^. This situation, added to precarious work process, lack of organizational resources, shift work, conflicting work relationships and low pay, further predisposes healthcare workers to conditions of distress and mental and physical illness, even affecting their quality of life (QoL)^(5,6)^.

This situation became even more complex with the COVID-19 pandemic, a public health concern that was related to increased prevalence of symptoms and mental disorders in healthcare professionals who provided close assistance to infected patients, especially nursing staff, which added to the context, previously vulnerable, fear of the unknown, feeling of unsafety, fear for one’s health and life as well as that of family and friends, in addition to the need for social isolation^(7,8)^. In this regard, one of the concerns was the impact of this scenario on nursing professionals’ QoL.

Taking into consideration the World Health Organization’s (WHO) concept of QoL as an individual’s perception of their position in life in the context of the culture and value systems, and relating them to their goals, expectations, standards and concerns, we considered the broad and comprehensive power of the construct, with a multidimensional essence, which systematically incorporates health, autonomy and the cultural, social and environmental context in which a person is inserted^(9,10)^.

Even before the pandemic, studies demonstrated that nursing professionals’ QoL should be maintained or improved, as in addition to impacting physical and mental exhaustion, it could affect their ability to work. The needs for satisfaction, safety, access to health, socio-environmental conditions, individual financial and self-care possibilities, in addition to institutional resources, organizational climate and relationships, are related to the entire context of the work required for this professional category, which can compromise overall QoL and not just at work^(11,12)^.

To implement improvements in workers’ QoL and health, monitoring capable of detecting and analyzing possible changes that impact these individuals’ well-being and health-disease process and how they relate to the work context is essential^(13,14)^. Given that QoL is mostly studied and analyzing people with chronic diseases, there is a considerable gap in studies that consider this topic and healthcare providers’ health^(15)^.

## OBJECTIVES

To assess the physical and mental components of QoL and associate them with nursing professionals’ individual, health and work characteristics in a tertiary teaching hospital.

## METHODS

### Ethical aspects

Confidentiality and voluntariness were guaranteed to participants, who signed the Informed Consent Form (ICF), and were informed about aspects related to the research. The research was approved by the *Universidade Federal de São Paulo* Research Ethics Committee.

### Study design, period and location

A cross-sectional study^(16)^ was carried out based on the STrengthening the Reporting of OBservational studies in Epidemiology (STROBE) framework from the EQUATOR network, in a tertiary teaching hospital, linked to a public university in São Paulo, SP.

### Population, sample and criteria

The population included nursing professionals (nurses, nursing technicians and assistants) who worked in direct and/or indirect care at the hospital and associated outpatient clinics, who had worked at the institution for more than a year and who were not on vacation or leave at the time of data collection. The minimum sample size of 277 people was obtained using a previously described formula when the population size is known^(17)^, for a finite population of 982 individuals. The sample was randomly selected and totaled 280 professionals.

### Study protocol

Data collection took place between May 2021 and February 2022. Contact with the selected individuals was carried out by telephone or email, at which time the study objectives and the voluntary nature of participation were clarified. Upon acceptance and digital signature of the ICF, participants were sent the link to access the questionnaires and instruments available on Google Forms®. Participants’ answers were automatically tabulated and converted into a database to avoid typing or tabulation errors. Except for the sociodemographic questionnaire, the order of the instruments was constantly changed.

QoL was assessed by the Medical Outcomes Study 36-item Short-Form Health Survey (SF-36), translated and validated for Brazil. This generic health assessment instrument contains 36 items, divided into eight dimensions, each with a different calculation that generates scores ranging from 0, the worst state, to 100, the best QoL state^(18)^. The synthesis scores of the physical components (physical functioning, role physical, bodily pain and general health) and mental components (role emotional, vitality, social functioning and mental health) were calculated^(8,19,20,21)^. Dimensional scores lower than 70 were considered “worst state” of QoL and equal to or greater than 70 “best state”^(22)^.

Individual and work variables were assessed using a sociode-mographic questionnaire developed specifically for this study.

Work ability (WA) was defined as how well workers are or will be in the near future and how capable they are of performing their job, and was assessed using the Work Ability Index (WAI)^(23,24)^, which presents a Cronbach’s alpha of 0.80 in nursing professionals^(25)^, good performance in relation to construct, reliability and criterion validity, in addition to satisfactory psychometric properties^(14,25)^. The analysis was based on the classification of scores as low WA for values from 7 to 27, moderate for 28 to 36, good for 37 to 43 and excellent for scores from 44 to 49^(23,24)^.

Organizational climate was defined as the common perception of individuals regarding the entire work context^(13,26)^, and was measured by the Organizational Climate Scale (OCS), which presented internal consistency of 0.78 to 0.92. The analysis of the factors “support of leader and organization”, “reward”, “physical comfort” and “cohesion among peer” considers that the higher the score, the better the organizational climate and values greater than 4 tend to indicate a climate good and lower than 2.9, poor climate. For the “control/pressure” factor, the values are inverse, i.e., values less than 2.9 indicate good climate and greater than 4, poor climate^(13)^.

Daytime sleepiness was characterized by the ability to remain awake and/or alert throughout the day^(27)^, assessed using the Epworth Sleepiness Scale (ESS), which indicates daily situations that can cause drowsiness. They are scored from 0 (would never doze) to 3 (high probability of dozing), with good reliability, internal consistency and validation for use in Brazil. The sum of the answers can generate a score from 0 to 24, values below 10 indicate low propensity to sleep, from 10 to 16 excessive daytime sleepiness and greater than 16, severe drowsiness^(28,29)^.

### Analysis of results, and statistics

The statistical program used in the descriptive and inferential analyzes was Jamovi®. Bivariate analysis was based on the MannWhitney and Kruskal-Wallis tests. Multiple linear regression was used in multivariate analysis, meeting the verified assumptions of the coefficient of determination R^2^ and adjusted R^2^, autocorrelation, test of normality of residuals, multicollinearity and variance.

## RESULTS

Participants were predominantly female nursing assistants, married, between 40 and 59 years old (Table 1).

**Table 1:** Distribution of the sample of nursing professionals according to individual characteristics, São Paulo, São Paulo, Brazil, 2023

Variable	Category	n (%)
Age group	Up to 39 years old	76 (27.1)
	40 – 49 years old	123 (43.9)
	50 – 59 years old	60 (21.5)
	60 or older	21 (7.5)
Gender	Female	236 (84.3)
	Male	44 (15.7)
Marital status	Married	161 (57.5)
	Single	69 (24.6)
	Separated/divorced/widowed	50 (17.9)
Family income	Between R$1,500.00 and R$4,500.00	67 (23.9)
	Between R$4,501.00 and R$6,000.00	61 (21.8)
	Between R$6,001.00 and R$10,000.00	91 (32.5)
	Above R$ 10,001.00	61 (21.8)
Weekly working hours	Up to 40 hours	157 (56.0)
	41 to 60 hours	105 (37.5)
	61 or more	18 (6.4)
Self-rated physical health	Good	131 (46.8)
	Average	122 (43.6)
	Poor	27 (9.6)
Self-rated mental	Good	142 (50.7)
	Average	108 (38.6)
	Poor	30 (10.7)
Satisfaction with work	Very satisfied	90 (32.2)
	Neutral	137 (48.9)
	Dissatisfied	53 (18.9)
Reports work-related illness	Yes	139 (49.6)
	No	141 (50.4)
Weekly frequency of physical activity	None	172 (61.4)
	1 - 2 times	40 (14.3)
	3 - 5 times	62 (22.2)
	6 - 7 times	6 (2.1)
Total		280 (100)

*n – absolute frequency; % – relative frequency.*

Most participants presented a poor organizational climate in most factors, a tendency towards low QoL in the physical and mental components and a little less than half of the WA sample was moderate or poor. Half of participants had excessive or severe daytime sleepiness (Table 2).

**Table 2:** Distribution of the sample of nursing professionals according to organizational climate, quality of life components and work ability, São Paulo, São Paulo, Brazil, 2023

Variable	Category	n (%) or X ± SD
Organizational climate (OCS)	Support of leader and organization	3.0 ± 0.7
	Reward	2.1 ± 0.6
	Physical comfort	2.8 ± 0.7
	Control/pressure	2.7 ± 0.6
	Cohesion among peers	3.6 ± 0.7
Quality of life (SF-36)	Physical component	67.93 ± 19.9
	Mental component	63.13 ± 23
Work ability (WAI)	Excellent	26 (9.3)
	Good	126 (45.0)
	Moderate	112 (40.0)
	Poor	16 (5.7)
Daytime sleepiness (ESS)	Low	141 (50.4)
	Excessive	93 (33.2)
	Severe	46 (16.4)
Total		280 (100)

*N – absolute frequency; % – relative frequency; X – average; SD – standard deviation; OCS > 4 – good climate and < 2.9 – poor climate; “control/pressure” factor, inverse score; SF-36 <70 – worst condition; > 70 – best condition; WAI 44-49 – excellent; 37-43 – good; 28-36 – moderate; 7-27 – low; ESS <10 – low; 10–16 – excessive; > 16 – severe.*

Table 3 shows that the physical component of the QoL domains worsens with the reduction in family income, self-report of work-related illnesses, decreased WA, greater control/pressure on workers and increased daytime sleepiness. On the other hand, it improves with an increase in the weekly frequency of physical activity and the perception of greater support of leader and organization.

**Table 3:** Association between individual and work-related characteristics and the physical component of quality of life domains, carried out through multiple linear regression, São Paulo, São Paulo, Brazil, 2023

Variable	Estimate	95% Confidence Interval		*p* value
		LL	UL	
Family income				
> R$ 10,001.00				
R$ 6,001.00-R$ 10,000.00	-3.1	-7.8	1.5	0.19
R$ 4,501.00-R$ 6,000.00	-4.0	-9.1	1.1	0.12
R$ 1,500.00– R$ 4,500.00	-10.7	-15.8	-5.7	< 0.001
Physical activity	1.8	0.8	2.7	< 0.001
Illness	-5.9	-9.6	-2.3	0.001
WAI				
Excellent				
Good	-7.1	-13.3	-1.0	0.02
Moderate	-21.2	-27.7	-14.6	< 0.001
Poor	-37.1	-46.9	-27.3	< 0.001
OCS				
Support of leader and organization	4.0	1.4	6.5	0.002
Control/pressure	-5.0	-8.0	-2.0	< 0.001
ESS				
Low				
Excessive	-0.3	-4.1	3.4	0.86
Severe	-5.6	-10.4	-0.8	0.02

*Model determination coefficient: R – 0.73; R^2^ – 0.53; Adjusted R^2^ – 0.51. Model met validity assumptions, no correlation between each other, adequate values in all variance inflation factors and residual normality test. WAI – Work Ability Index: 44-49 – excellent; 37-43 – good; 28-36 – moderate; 7-27 – low; OCS – Organizational Climate Scale: > 4 – good climate; < 2.9 – poor climate; “control/pressure” factor, inverse score; ESS – Epworth Sleepiness Scale: < 10 – low; 10–16 – excessive; > 16 – severe. LL – lower limit; UL – upper limit.*

In relation to the mental component of the QoL domains, improvements are observed with increasing age and worsening with reduced job satisfaction, self-report of work-related illnesses, lower self-rated physical health, reduced WA and increased daytime sleepiness (Table 4).

**Table 4:** Association between individual and work-related characteristics and the mental component of quality of life domains, carried out through multiple linear regression, São Paulo, São Paulo, Brazil, 2023

Variable	Estimate	95% Confidence Interval		*p* value
		LL	UL	
Age range				
Up to 39 years old				
40 – 49 years old	8.2	2.8	13.5	0.003
50 – 59 years old	8.9	2.4	15.5	0.007
60 years or older	9.0	-0.0	18.2	0.052
Job satisfaction				
Very satisfied				
Neutral	-6.2	-11.3	-1.2	0.015
Dissatisfied	-11.3	-18.0	-4.6	<0.001
Report work-related illness	-5.9	-10.6	-1.1	0.016
Self-rated physical health				
Good				
Average	-6.4	-11.3	-1.6	0.009
Poor	-15.9	-24.6	-7.2	<0.001
WAI				
Excellent				
Good	-13.9	-22.0	-5.7	<0.001
Moderate	-20.5	-29.5	-11.6	<0.001
Poor	-31.0	-44.6	-17.4	<0.001
ESS				
Low				
Excessive	-1.8	-6.8	3.1	0.46
Severe	-9.8	-16.1	-3.5	0.002

*Model determination coefficient: R – 0.63; R^2^ – 0.39; Adjusted R^2^ – 0.37. Model met validity assumptions, no correlation between each other, adequate values in all variance inflation factors and residual normality test. WAI – Work Ability Index: 44-49 – excellent; 37-43 – good; 28-36 – moderate; 7-27 – low; ESS – Epworth Sleepiness Scale: < 10 – low; 10–16 – excessive; > 16 – severe. LL – lower limit; UL – upper limit.*

## DISCUSSION

Worsening in the physical component of QoL domains was related to reduced family income, self-reporting of work-related illnesses, decreased WA, greater control/pressure on workers and increased daytime sleepiness. Higher scores in this component were related to increased weekly frequency of physical activity and greater support of leader and organization. Regarding the mental component, higher scores were related to increasing age, and worsening was related to reduced job satisfaction, self-reported work-related illnesses, worse self-rated physical health, reduced WA and increased daytime drowsiness.

The sociodemographic profile of the studied sample supports that described in other studies with nursing professionals, in which individuals were mostly under 50 years old^(30,31)^, female^(30,32,33)^, married or living in a stable union^(32,33)^; however, the family income found was higher than in other Brazilian studies^(31,34)^.

In the regression analysis, the QoL mental component was associated with age group, with progressive worsening as age increased. Before the pandemic, this variable was already related to QoL in all domains, indicating that younger people tend to have less impaired QoL, which is also associated with better WA. Younger professionals have less occurrence of chronic pain, need for medical treatment and are more willing to carry out daily activities^(35,36,37)^.

Paid and unpaid work workload did not impact these professionals’ QoL, unlike what has been described in other studies, where increased working hours significantly impacted QoL, especially considering that, during the pandemic, working hours may have been substantially higher than usual^(30,36)^.

During the pandemic, the general population’s QoL was affected, with a drastic impact on the social component, due to recommended social distancing, which required adaptations and greater support from technology in maintaining bonds and connections with family and friends, with social support being a significant factor for the adequate QoL of healthcare professionals^(20,38)^. However, the low QoL found in the present study reinforces that nursing professionals, even before the pandemic, already showed regular or poor perception in the QoL physical, psychological and environmental domains, in addition to changes in WA, sleep and rest, fatigue and cognitive aspects, factors statistically associated with work overload, lack of human and material resources, inadequate physical structure, stress, low wages, disorganized environment, poor interpersonal relationships with colleagues, patients and managers, lack of social support, lack of physical activity and leisure, irregular sleep and lack of safety^(31,35,37,39,40,41)^.

On the other hand, a study carried out with nursing professionals during the pandemic showed that, despite the high level of stress and damage to the QoL mental component scores, there were good scores regarding physical aspects and high scores for self-efficacy and resilience^(30)^. In the present study, the mental component synthesis was the most affected and this included the score relating to social aspects, in line with what has been described in other studies. This aspect of QoL assesses the social, sexual and support circle of family and friends. Despite the undeniable impact of the pandemic on healthcare professionals, nursing was already facing difficulties, such as shift work, often incompatible with that of family and friends, limiting their participation in social activities, physical activities and impacting their time available for sleep and rest^(8,34)^.

The lack of policies that consider QoL in hospital organization stands out, which is a fundamental discussion currently^(41)^. For nursing professionals, exposed to a stressful context of overload and exposure to factors that compromise health in the work environment^(42)^, QoL demands special care, especially due to labor vulnerability and the impact generated on the care provided, and should be monitored more frequently, making it possible to plan more and better actions related to improving workers’ QoL^(39,40,41,42)^, such as programs that develop skills and competencies focused on the combination of self-efficacy and resilience^(30)^. Mental resources, social support, happiness and job satisfaction were associated with QoL before and during the pandemic^(30,32,37)^, showing that protecting healthcare professionals’ mental health must be an imperative condition of Brazilian strategies in the post-pandemic period, when professionals will continue to act fundamentally in facing physical and mental rehabilitation’ needs as well as in meeting all the health demands contained in periods of social isolation and low access to health for events unrelated to COVID^(7)^.

Regression analysis demonstrated an association with family income, and, below R$4,500.00, the QoL physical component showed a worsening of 10.79 points, which supported other national and international findings, in which general QoL, at work and in environmental and psychological aspects were affected by income before and during the pandemic. Low or insufficient remuneration is a historical fact in the field of nursing, currently being discussed in various spheres^(8,31,34,35)^.

Each added day of physical activity improved QoL in the physical component by 1.83 points, a result similar to that in the literature, including in the pandemic, when the low frequency of physical activity combined with stress showed a direct association with professionals’ QoL and health^(8,34)^. It has been proven that practicing physical activity benefits health with immediate and long-term effects, which also favors the control of chronic diseases and comorbidities^(38)^.

Concerning some type of work-related illness affected QoL in the physical and mental components, according to other national and international findings, regardless of the period^(8,36,37,38)^. Likewise, WA was associated with both components of QoL, with worsening of WA being the most important aspect identified in the association, as poor WA worsened QoL by 37.14 points in the physical component and by 31.06 points in the mental component, as also described in the literature^(37)^.

The worse the self-rated health, the worse the QoL score in the mental component. The way a person self-rates their own physical and mental health is of fundamental importance when making decisions about their self-care, as it favors the adoption of disease prevention and health promotion habits that can reduce risks and vulnerabilities. A poor self-rated physical health can have important impacts on the QoL mental component, since emotions can play a significant role in physical health and vice versa. The limitations resulting from this self-assessment can make it difficult to participate in activities that were previously enjoyable, leading to feelings of isolation, loneliness, inadequacy, low self-esteem, hopelessness and sadness, creating a negative cycle that affects mental health and in turn worsens physical symptoms^(43)^.

In the present study, WA was classified as good or excellent in just over half of participants, while in 45.7% it was moderate or poor, relatively worse data than those found in other studies carried out with nursing professionals^(44,45)^. In these professionals, WA has been identified as a fundamental indicator to monitor and enable early intervention in aspects of worker health, considering the internal physical and mental dimension and external aspects related to working conditions, resulting in improvements in QoL and boosting the organization’s overall productivity^(37,46)^.

Daytime drowsiness is an aspect that has been shown to be particularly important in this study as well as in others involving nursing professionals, as daytime drowsiness and insomnia symptoms rates have been alarming and have been shown to be significantly associated with QoL. Better QoL scores, in all domains, have previously been associated with average sleep time^(34,47,48)^. The challenge is to adapt sleep to nursing professionals’ rhythm of life and work, considering their vulnerability to risk, frequent fatigue and the need to always be attentive and make quick decisions under pressure as a consequence of the characteristics of work. In addition to the harm to occupational health, excessive daytime sleepiness is directly associated with exposure to risks of errors and accidents at work, and this means increased risk of morbidity and mortality for themselves and for service users^(47,48)^.

In the present study, almost half of participants considered themselves neutral in relation to job satisfaction, lower than that found by other authors^(26,32)^. Poor organizational climate, in most factors, was in line with other findings involving nursing professionals^(26,49)^. Multiple linear regression analysis also demonstrated a significant statistical association between job satisfaction and the mental component of QoL, whereas organizational climate, in support of leader and organization and control/pressure factors, significantly impacted the physical component.

Studies have sought to understand how nursing workers’ perception of their work contexts and their behavior in organizations take into account the positive and negative aspects of the work environment and their influence on satisfaction, motivation, well-being and quality of the service provided. This analysis is particularly important in nursing so that it is possible to understand how workers are affected by working conditions, relationships with colleagues, the company and managers, interpersonal communication, benefits and rewards, as well as statistically relating the organizational climate dimensions with job satisfaction, motivation to work, professional turnover intention, among others^(26,32,37,42,49)^.

A study carried out with nurses in Iran, before the pandemic, reported an improvement in QoL when there was increased support from the institution’s superiors, reduced pressure in the work environment, development of programs that improved happiness and vitality and that were able to develop, in the community, a positive mindset towards the profession and understanding of the physical and mental problems faced by workers^(32)^. At the same time, a Canadian study, carried out with nursing professionals, showed that improvements in the work environment and employment status improved the care provided to patients and professionals’ health-related QoL. Furthermore, improvements in the work environment, better human and material resources and a lower workload were positively related to the QoL of those assessed. In the aforementioned study, it was suggested that it is more meaningful to focus on improving the work environment than on individual factors. Although personal resilience is a predictor of QoL, work factors such as resource adequacy had a greater impact^(21)^.

Improvements in people’s QoL can be achieved by investing in increasing professional satisfaction, motivation at work, freedom of expression within the service, pride in the profession, better working conditions, benefits offered by the company, equal treatment of employees, training and learning environment, level of participation in decisions, level of responsibility, interpersonal relationships, open and honest communication, clarity of roles, reduction of pressure on workers, minimization of stress and investment in increasing WA^(31,37)^.

### Study limitations

This study has limitations related to the cross-sectional design and sample, which was restricted to nursing professionals, who are public agents from a single institution.

### Contributions to nursing

Identifying and addressing these factors has implications for all nursing professionals and the institution, since being aware of one’s own QoL is a condition without which there is no mobilization to seek improvements. Leaders must recognize the needs of their team and seek to promote harmony and balance in relationships at work. The institution needs to recognize the importance of the QoL of its professionals so that it can offer a better working environment, with adequate policies and resources, in addition to being able to organize work flows so that individuals develop and apply their personal and professional skills for the benefit of the population they assist.

## CONCLUSIONS

QoL was considered low for nursing professionals in the analyzed sample. The physical component was inversely associated with family income. The organizational climate in the “control/pressure” factor also affected physical QoL. However, physical activity and better support of leader and organization resulted in improvements in this regard. The mental component was harmed by low job satisfaction and poor self-rated physical health. On the other hand, it was better evaluated by younger professionals. Both components of QoL worsened when nursing professionals reported work-related illnesses, low WA and worsening daytime sleepiness.
