# Supplementary material for: Quality of life associated with nursing professionals’ individual resources and work
Source: Rev Bras Enferm. 2024 Jun 17;77(3):e20230476. doi: 10.1590/0034-7167-2023-0476 (PMC11185074; doi:10.1590/0034-7167-2023-0476)
Supplement: Translation

# Translation: Qualidade de vida associada a recursos individuais e do trabalho de profissionais de enfermagem

**DOI:** 10.1590/0034-7167-2023-0476pt

**Authors:** Isabela Saura Sartoreto Mallagoli, Evandro Piccinelli da Silva, Maria Aline do Nascimento Oliveira, Italo Everton Bezerra Barbosa, Aristeia Nunes Sampaio, Aline Bicalho Matias, Dulce Aparecida Barbosa, Angélica Gonçalves Silva Belasco

**Affiliations:** I Universidade Federal de São Paulo. São Paulo, São Paulo, Brasil; II Universidade Federal do Acre. Rio Branco, Acre, Brasil

**Keywords:** Qualidade de Vida, Enfermagem, Saúde Ocupacional, Saúde Mental, Profissionais de Enfermagem

## Abstract

**Objetivos:**

avaliar os componentes físico e mental da qualidade de vida de profissionais de enfermagem e associá-los às características individuais, de saúde e do trabalho.

**Métodos:**

pesquisa transversal, com profissionais de enfermagem de um hospital universitário de São Paulo. Aplicados questionário próprio e instrumentos validados.

**Resultados:**

a qualidade de vida geral mostrou-se comprometida. O componente físico foi menor em relação à baixa renda familiar e entre os que percebiam maior controle/pressão no trabalho, e melhor para quem praticava atividade física e tinha apoio da chefia e organização. O componente mental foi menor nos profissionais que referiram insatisfação com o trabalho, pior autoavaliação de saúde física e com idade elevada. Escores de ambos componentes reduziram mediante doenças relacionadas ao trabalho, pior capacidade para o trabalho e aumento da sonolência diurna.

**Conclusões:**

a qualidade de vida foi estatisticamente associada a fatores institucionais controláveis e recursos individuais que, exceto idade, podem ser promovidos.

## INTRODUÇÃO

A enfermagem é um elemento fundamental em todo o sistema de saúde, representando em torno de 59% da força de trabalho em saúde do mundo^(1)^. No Brasil, dados do Conselho Federal de Enfermagem consideram a existência de mais de 2,8 milhões desses profissionais, dos quais 25,66% estão só no estado de São Paulo^(2)^.

O contato diário dos profissionais de enfermagem com situações de níveis variados de complexidade, múltiplas condições de desproteção e riscos à saúde favorece o desenvolvimento de disfunções físicas e mentais^(3,4)^. Esse quadro, somado à precarização do processo de trabalho, falta de recursos organizacionais, trabalho em turnos, relações de trabalho conflituosas e baixa remuneração, predispõe ainda mais o trabalhador de saúde a condições de sofrimento e adoecimento mental e físico, afetando inclusive sua qualidade de vida (QV)^(5,6)^.

Essa situação ficou ainda mais complexa com a pandemia de COVID-19, problema de saúde pública que foi relacionado ao aumento da prevalência de sintomas e transtornos mentais em profissionais de saúde que prestavam assistência próxima aos pacientes infectados, especialmente equipe de enfermagem, que somou ao contexto, antes vulnerável, o medo do desconhecido, a sensação de insegurança, o temor pela saúde e vida própria, como a dos familiares e amigos, além da necessidade de isolamento social^(7,8)^. Nesse sentido, uma das preocupações foi o impacto desse cenário na QV dos profissionais de enfermagem.

Levando em consideração o conceito de QV da Organização Mundial da Saúde (OMS) como a percepção individual da própria vida, contexto cultural e sistema de valores, e relacionando-os aos seus objetivos, expectativa, padrões e preocupações, consideramos a potência ampla e abrangente do construto, de essência multidimensional, que incorpora, de forma sistêmica, a saúde, a autonomia e o contex to cultural, social e ambiental no qual a pessoa está inserida^(9,10)^.

Mesmo antes da pandemia, estudos demonstraram que a QV de profissionais de enfermagem deveria ser mantida ou melhorada, pois, além de impactar o desgaste físico e mental, poderia afetar a capacidade para o trabalho. As necessidades de satisfação, segurança, acesso à saúde, condições socioambientais, possibilidades individuais financeiras e de autocuidado, além de recursos institucionais, clima e relacionamento organizacionais, relacionam-se com todo o contexto do trabalho exigido para essa categoria profissional, o que pode comprometer a QV de maneira geral e não apenas no trabalho^(11,12)^.

Para implementar melhorias na QV e saúde dos trabalhadores, é fundamental um monitoramento capaz de detectar e analisar possíveis alterações que impactem o bem-estar e o processo de saúde-doença desses indivíduos e como se relacionam ao contexto do trabalho^(13,14)^. Dado que a QV é majoritariamente estudada e analisando portadores de doenças crônicas, nota-se uma lacuna considerável de estudos que considerem este tema e a saúde dos prestadores de serviços de saúde^(15)^.

## OBJETIVOS

Avaliar os componentes físico e mental da QV e associá-los às características individuais, de saúde e de trabalho dos profissionais da enfermagem de um hospital terciário de ensino.

## MÉTODOS

### Aspectos éticos

O sigilo e a voluntariedade foram garantidos aos participantes, que assinaram o Termo de Consentimento Livre e Esclarecido (TCLE), e foram esclarecidos quanto aos aspectos relacionados à pesquisa. A pesquisa foi aprovada pelo Comitê de Ética e Pesquisa da Universidade Federal de São Paulo.

### Desenho, período e local do estudo

Foi realizado estudo transversal^(16)^ com base no referencial *STrengthening the Reporting of OBservational studies in Epidemiology* (STROBE) da rede EQUATOR, em hospital terciário de ensino, vinculado a uma universidade pública em São Paulo, SP.

### População, amostra e critérios

A população incluiu profissionais de enfermagem (enfermeiros, técnicos e auxiliares de enfermagem) que atuavam na assistência direta e/ou indireta no hospital e ambulatórios associados, que trabalhavam há mais de um ano na instituição e que não estavam de férias ou licença no momento da coleta de dados. O tamanho amostral mínimo de 277 pessoas foi obtido, através de fórmula previamente descrita quando o tamanho da população é conhecido^(17)^, para uma população finita de 982 indivíduos. A amostra foi selecionada aleatoriamente e totalizou 280 profissionais.

### Protocolo do estudo

A coleta de dados ocorreu entre maio de 2021 e fevereiro de 2022. O contato com os indivíduos selecionados foi realizado por telefone ou e-mail, momento em que eram esclarecidos os objetivos do estudo e a voluntariedade da participação. A partir do aceite e assinatura digital do TCLE, foi encaminhado ao participante o *link* para acesso aos questionários e instrumentos, disponíveis na ferramenta *Google Forms®.* As respostas dos participantes foram automaticamente tabuladas e convertidas em banco de dados, para evitar erros de digitação ou tabulação. Exceto para o questionário sociodemográfico, a ordem dos instrumentos, foi alterada constantemente.

A QV foi avaliada pelo questionário *Medical Outcomes Study 36-item Short-Form Health Survey* (SF-36), traduzido e validado para o Brasil. Esse instrumento genérico de avaliação de saúde contém 36 itens, divididos em oito dimensões, cada uma com um cálculo diferenciado que gera escores que variam de 0, pior estado, a 100, melhor estado da QV^(18)^. Foram calculadas as pontuações dos resumos dos componentes físicos (capacidade funcional, aspectos físicos, dor e estado geral de saúde) e mentais (aspectos emocionais, vitalidade, aspectos sociais e saúde mental)^(8,19,20,21)^. Escores das dimensões inferiores a 70 foram considerados como “pior estado” de QV, e iguais ou superiores a 70, como “melhor estado”^(22)^.

Variáveis individuais e do trabalho foram avaliadas a partir de um questionário sociodemográfico desenvolvido especificamente para este estudo.

Capacidade para o trabalho (CT) foi definida como quão bem está ou estará o trabalhador em um futuro próximo e quão capaz de realizar seu trabalho ele é, e foi avaliada através do Índice de Capacidade para o Trabalho (ICT)^(23,24)^, que apresenta alfa de Cronbach de 0,80 em profissionais de enfermagem^(25)^, bom desempenho com relação à validade de construto, de confiabilidade e de critério, além de propriedades psicométricas satisfatórias^(14,25)^. A análise foi baseada na classificação dos escores em CT baixa para valores de 7 a 27, moderada para 28 a 36, boa para 37 a 43 e ótima para pontuações de 44 a 49^(23,24)^.

Clima organizacional foi definido como a percepção comum dos indivíduos com relação a todo o contexto laboral^(13,26)^, e foi medido pela Escala de Clima Organizacional (ECO), que apresentou consistência interna de 0,78 a 0,92. A análise dos fatores “apoio da chefia e da organização”, “recompensa”, “conforto físico” e “coesão entre colegas” considera que, quanto maior o valor da pontuação, melhor o clima organizacional, e valores maiores que 4 tendem a indicar um clima bom, e menores que 2,9, um clima ruim. Para o fator “controle/pressão”, os valores são inversos, ou seja, valores menores que 2,9 indicam clima bom, e maiores que 4, clima ruim^(13)^.

Sonolência diurna foi caracterizada pela capacidade de se manter acordado e/ou alerta ao longo do dia^(27)^, avaliada através da Escala de Sonolência de Epworth (ESE), que indica situações diárias que podem gerar sonolência. São pontuadas de 0 (nunca cochilaria) a 3 (grande probabilidade de cochilar), com boa confiabilidade, consistência interna e validação para uso no Brasil. A soma das respostas pode gerar uma pontuação de 0 a 24, sendo que valores inferiores a 10 indicam propensão baixa para dormir, de 10 a 16, sonolência diurna excessiva, e maior que 16, sonolência grave^(28,29)^.

### Análise dos resultados e estatística

O programa estatístico utilizado nas análises descritivas e inferenciais foi o *Jamovi*®. A análise bivariada foi pautada nos Testes de Mann-Whitney e Kruskal-Wallis. A regressão linear múltipla foi utilizada na análise multivariada, atendendo aos pressupostos verificados do coeficiente de determinação R^2^ e R^2^ ajustado, autocorrelação, teste de normalidade dos resíduos, multicolinearidade e variância.

## RESULTADOS

Os participantes eram predominantemente auxiliares de enfermagem, do gênero feminino, casadas e entre 40 e 59 anos (Tabela 1).

**Tabela 1:** Distribuição da amostra de profissionais de enfermagem segundo características individuais, São Paulo, São Paulo, Brasil, 2023

Variável	Categoria	n (%)
Faixa etária	Até 39 anos	76 (27,1)
	40 – 49 anos	123 (43,9)
	50 – 59 anos	60 (21,5)
	60 ou mais	21 (7,5)
Gênero	Feminino	236 (84,3)
	Masculino	44 (15,7)
Estado civil	Casado	161 (57,5)
	Solteiro	69 (24,6)
	Separado/divorciado/viúvo	50 (17,9)
Renda familiar	Entre R$ 1.500,00 e R$ 4.500,00	67 (23,9)
	Entre R$ 4.501,00 e R$ 6.000,00	61 (21,8)
	Entre R$ 6.001,00 e R$ 10.000,00	91 (32,5)
	Acima de R$ 10.001,00	61 (21,8)
Jornada de trabalho semanal	Até 40 horas	157 (56,0)
	41 a 60 horas	105 (37,5)
	61 ou mais	18 (6,4)
Autoavaliação da saúde física	Boa	131 (46,8)
	Média	122 (43,6)
	Ruim	27 (9,6)
Autoavaliação da saúde mental	Boa	142 (50,7)
	Média	108 (38,6)
	Ruim	30 (10,7)
Satisfação com o trabalho	Muito satisfeito	90 (32,2)
	Neutro	137 (48,9)
	Insatisfeito	53 (18,9)
Refere doença relacionada ao trabalho	Sim	139 (49,6)
	Não	141 (50,4)
Frequência semanal de atividade física	Nenhuma	172 (61,4)
	1 - 2 vezes	40 (14,3)
	3 - 5 vezes	62 (22,2)
	6 - 7 vezes	6 (2,1)
Total		280 (100)

*n – frequência absoluta; % – frequência relativa.*

A maior parte dos participantes apresentou clima organizacional ruim na maioria dos fatores, tendência à baixa QV nos componentes físico e mental e um pouco menos da metade da amostra CT moderada ou ruim. Metade dos participantes apresentou sonolência diurna excessiva ou grave (Tabela 2).

**Tabela 2:** Distribuição da amostra de profissionais de enfermagem segundo clima organizacional, componentes de qualidade de vida e capacidade para o trabalho, São Paulo, São Paulo, Brasil, 2023

Variável	Categoria	n (%) ou X ± DP
Clima organizacional (ECO)	Apoio da chefia e da organização	3,0 ± 0,7
	Recompensa	2,1 ± 0,6
	Conforto físico	2,8 ± 0,7
	Controle/pressão	2,7 ± 0,6
	Coesão entre colegas	3,6 ± 0,7
Qualidade de vida (SF-36)	Componente físico	67,93 ± 19,9
	Componente mental	63,13 ± 23
Capacidade para o trabalho (ICT)	Ótima	26 (9,3)
	Boa	126 (45,0)
	Moderada	112 (40,0)
	Ruim	16 (5,7)
Sonolência diurna (ESE)	Baixa	141 (50,4)
	Excessiva	93 (33,2)
	Grave	46 (16,4)
Total		280 (100)

*n – frequência absoluta; % – frequência relativa; X – média; DP – desvio padrão; ECO > 4 – clima bom e < 2,9 – clima ruim; fator “controle/pressão” pontuação inversa; SF-36 < 70 – pior estado; > 70 – melhor estado; ICT 44-49 – ótima; 37-43 – boa; 28-36 – moderada; 7-27 – baixa; ESE < 10 – baixa; 10–16 – excessiva; > 16 – grave.*

Na Tabela 3, observa-se que o componente físico dos domínios de QV piora com a redução da renda familiar, autorrelato de doenças relacionadas ao trabalho, diminuição da CT, maior controle/pressão sobre o trabalhador e aumento da sonolência diurna. Por outro lado, melhora com o aumento da frequência semanal de atividade física e percepção de maior apoio da chefia e da organização.

**Tabela 3:** Associação entre características individuais e relacionadas ao trabalho e o componente físico dos domínios de qualidade de vida, realizada através da regressão linear múltipla, São Paulo, São Paulo, Brasil, 2023

Variável	Estimativa	Intervalo de Confiança 95%		Valor de *p*
		LI	LS	
Renda familiar				
> R$ 10.001,00				
R$ 6.001,00-R$ 10.000,00	-3,1	-7,8	1,5	0,19
R$ 4.501,00-R$ 6.000,00	-4,0	-9,1	1,1	0,12
R$ 1.500,00– R$ 4.500,00	-10,7	-15,8	-5,7	< 0,001
Atividade física	1,8	0,8	2,7	< 0,001
Doença	-5,9	-9,6	-2,3	0,001
ICT				
Ótima				
Boa	-7,1	-13,3	-1,0	0,02
Moderada	-21,2	-27,7	-14,6	< 0,001
Ruim	-37,1	-46,9	-27,3	< 0,001
ECO				
Apoio da chefia e da organização	4,0	1,4	6,5	0,002
Controle/pressão	-5,0	-8,0	-2,0	< 0,001
ESE				
Baixa				
Excessiva	-0,3	-4,1	3,4	0,86
Grave	-5,6	-10,4	-0,8	0,02

*Coeficiente de determinação do modelo: R – 0,73; R^2^ – 0,53; R^2^ ajustado – 0,51. Modelo atendeu a pressupostos de validade, sem correlação entre si, valores adequados em todos os fatores de inflação da variância e teste de normalidade dos resíduos. ICT – Índice de Capacidade para o Trabalho: 44-49 – ótima; 37-43 – boa; 28-36 – moderada; 7-27 – baixa; ECO – Escala de Clima Organizacional: > 4 – clima bom; < 2,9 – clima ruim; fator “controle/pressão” pontuação inversa; ESE – Escala de Sonolência de Epworth: < 10 – baixa; 10–16 – excessiva; > 16 – grave. LI – limite inferior; LS – limite superior.*

Em relação ao componente mental dos domínios de QV, observa-se melhora quanto maior a idade e piora com a redução da satisfação no trabalho, autorrelato de doenças relacionadas ao trabalho, menor autoavaliação da saúde física, redução da CT e aumento da sonolência diurna (Tabela 4).

**Tabela 4:** Associação entre características individuais e relacionadas ao trabalho e o componente mental dos domínios de qualidade de vida, realizada através da regressão linear múltipla, São Paulo, São Paulo, Brasil, 2023

Variável	Estimativa	Intervalo de Confiança 95%		Valor de *p*
		LI	LS	
Faixa etária				
Até 39 anos				
40 – 49 anos	8,2	2,8	13,5	0,003
50 – 59 anos	8,9	2,4	15,5	0,007
60 anos ou mais	9,0	-0,0	18,2	0,052
Satisfação no trabalho				
Muito satisfeito				
Neutro	-6,2	-11,3	-1,2	0,015
Insatisfeito	-11,3	-18,0	-4,6	<0,001
Referir doença relacionada ao trabalho	-5,9	-10,6	-1,1	0,016
Autoavaliação da saúde física				
Boa				
Média	-6,4	-11,3	-1,6	0,009
Ruim	-15,9	-24,6	-7,2	<0,001
ICT				
Ótima				
Boa	-13,9	-22,0	-5,7	<0,001
Moderada	-20,5	-29,5	-11,6	<0,001
Ruim	-31,0	-44,6	-17,4	<0,001
ESE				
Baixa				
Excessiva	-1,8	-6,8	3,1	0,46
Grave	-9,8	-16,1	-3,5	0,002

*Coeficiente de determinação do modelo: R – 0,63; R2 – 0,39; R2 ajustado – 0,37. Modelo atendeu a pressupostos de validade, sem correlação entre si, valores adequados em todos os fatores de inflação da variância e teste de normalidade dos resíduos. ICT – Índice de Capacidade para o Trabalho: 44-49 – ótima; 37-43 – boa; 28-36 – moderada; 7-27 – baixa; ESE – Escala de Sonolência de Epworth: < 10 – baixa; 10–16 – excessiva; > 16 – grave. LI – limite inferior; LS – limite superior.*

## DISCUSSÃO

Piora no componente físico dos domínios de QV foi relacionado à redução da renda familiar, ao autorrelato de doenças relacionadas ao trabalho, à diminuição da CT, ao maior controle/ pressão sobre o trabalhador e ao aumento da sonolência diurna. Maiores pontuações nesse componente foram relacionadas ao aumento da frequência semanal de atividade física e ao maior apoio da chefia e da organização. Em relação ao componente mental, maiores pontuações foram relacionadas ao aumento da idade, e a piora foi relacionada à redução da satisfação no trabalho, ao autorrelato de doenças relacionadas ao trabalho, à pior autoavaliação da saúde física, à redução da CT e ao aumento da sonolência diurna.

O perfil sociodemográfico da amostra estudada corrobora o descrito em outros estudos com profissionais de enfermagem, em que os indivíduos tinham majoritariamente idade inferior a 50 anos^(30,31)^, eram do gênero feminino^(30,32,33)^, casados ou vivendo em união estável^(32,33)^, entretanto a renda familiar encontrada foi superior a de outros estudos brasileiros^(31,34)^.

Na análise de regressão, o componente mental da QV foi associado à faixa etária, com piora progressiva conforme aumento da idade. Antes da pandemia, essa variável já era relacionada à QV em todos os domínios, indicando que pessoas mais jovens tendem a ter a QV menos prejudicada, o que também está associado a melhor CT. Profissionais mais jovens têm menor ocorrência de dores crônicas, necessidade de tratamento médico e mais disposição para as atividades cotidianas^(35,36,37)^.

A carga horária de trabalho remunerado e não remunerado não impactou a QV desses profissionais, diferentemente do descrito em outros estudos, onde o aumento da carga horária impactou significativamente a QV, principalmente considerando que, durante a pandemia, a carga horária trabalhada pode ter sido substancialmente superior à habitual^(30,36)^.

Durante a pandemia, a QV da população geral foi afetada, com impacto drástico no componente social, devido ao distanciamento social indicado, que exigiu adaptações e maior apoio da tecnologia na manutenção de vínculos e conexão com familiares e amigos, sendo o suporte social um fator significativo para a adequada QV de profissionais de saúde^(20,38)^. Entretanto, a baixa QV encontrada no presente estudo reforça que profissionais de enfermagem, mesmo antes da pandemia, já mostravam percepção regular ou ruim nos domínios físico, psicológico e meio ambiente da QV, além da alteração na CT, sono e repouso, fadiga e aspectos cognitivos, fatores associados estatisticamente à sobrecarga de trabalho, à falta de recursos humanos e materiais, à estrutura física inadequada, ao estresse, aos baixos salários, à desorganização do ambiente, ao relacionamento interpessoal ruim com colegas, pacientes e chefias, à falta de apoio social, à falta de atividade física e de lazer, ao sono irregular e à falta de segurança^(31,35,37,39,40,41)^.

Por outro lado, estudo realizado com profissionais de enfermagem durante a pandemia mostrou que, apesar do alto nível de estresse e prejuízo aos escores do componente mental da QV, houve bons escores com relação aos aspectos físicos e elevadas pontuações de autoeficácia e resiliência^(30)^. No presente estudo, o resumo do componente mental foi o mais prejudicado e este abrangeu a pontuação referente aos aspectos sociais, indo ao encontro do descrito por outros estudos. Esse aspecto da QV avalia o círculo social, sexual e de apoio de familiares e amigos. Em que pese o impacto inegável da pandemia em profissionais de saúde, a enfermagem já enfrentava dificuldades, como trabalho em turnos, muitas vezes incompatíveis com os da família e amigos, limitando sua participação em atividades sociais, na prática de atividades físicas e impactando seu tempo disponível para sono e repouso^(8,34)^.

Destaca-se a carência de políticas que considerem a QV na organização hospitalar, sendo essa uma discussão fundamental atualmente^(41)^. Para os profissionais de enfermagem, expostos a um contexto estressante de sobrecarga e exposição a fatores que comprometem a saúde no ambiente de trabalho^(42)^, a QV demanda especial cuidado, sobretudo pela vulnerabilidade laboral e impacto gerado na assistência prestada, devendo ser mais frequentemente monitorada, possibilitando planejar mais e melhores ações relacionadas à melhoria da QV do trabalhador^(39,40,41,42)^, como programas que desenvolvam habilidade e competências centradas na combinação entre autoeficácia e resiliência^(30)^. Recursos mentais, suporte social, felicidade e satisfação no trabalho foram associados à QV antes e durante a pandemia^(30,32,37)^, mostrando que a proteção da saúde mental dos profissionais de saúde deve ser condição imperativa das estratégias brasileiras no período pós-pandêmico, quando os profissionais continuarão atuando fundamentalmente no enfrentamento das necessidades de reabilitação física e mental, bem como no atendimento de toda a demanda de saúde represada nos períodos de isolamento social e baixo acesso à saúde para os eventos alheios à COVID^(7)^.

A análise de regressão demonstrou associação com a renda familiar, sendo que, abaixo de R$ 4.500,00, o componente físico da QV apresentou piora de 10,79 pontos, o que corroborou outros achados nacionais e internacionais, nos quais a QV geral, no trabalho e nos aspectos ambientais e psicológicos foi afetada pela renda antes e durante a pandemia. A remuneração baixa ou insuficiente configura-se fato histórico no campo da enfermagem, sendo pauta de discussões em diversas esferas atualmente^(8,31,34,35)^.

Cada dia acrescentado de atividade física, melhorou a QV no componente físico em 1,83 pontos, resultado semelhante ao da literatura, inclusive na pandemia, quando a baixa frequência de atividade física aliada ao estresse apresentou associação direta na QV e na saúde dos profissionais^(8,34)^. Já foi provado que a prática de atividade física beneficia a saúde com efeitos imediatos e a longo prazo, o que também favorece o controle de doenças crônicas e comorbidades^(38)^.

Referir algum tipo de doença relacionada ao trabalho afetou a QV nos componentes físico e mental, conforme outros achados nacionais e internacionais, independentemente do período^(8,36,37,38)^. Do mesmo modo, a CT esteve associada a ambos os componentes da QV, sendo que a piora da CT foi o aspecto mais importante identificado na associação, pois a CT ruim piorou a QV em 37,14 pontos no componente físico e em 31,06 pontos no componente mental, como também descrito na literatura^(37)^.

Quanto pior a autoavaliação de saúde, pior o escore de QV no componente mental. A forma como a pessoa autoavalia sua própria saúde física e mental é de fundamental importância na tomada de decisão sobre seu autocuidado, pois favorece a adoção de hábitos de prevenção de doenças e promoção da saúde que pode reduzir riscos e vulnerabilidades. Uma autoavaliação ruim de saúde física pode ter impactos importantes no componente mental da QV, uma vez que emoções podem desempenhar papel significativo na saúde física e vice-versa. As limitações decorrentes dessa autoavaliação podem dificultar a participação em atividades que antes eram prazerosas, levando a sentimentos de isolamento, solidão, inadequação, baixa autoestima, desesperança e tristeza, criando um ciclo negativo que afeta a saúde mental e, por sua vez, agrava os sintomas físicos^(43)^.

No presente estudo, a CT foi classificada como boa ou ótima em pouco mais da metade dos participantes, enquanto que, em 45,7%, mostrou-se moderada ou ruim, dado relativamente pior do que os encontrados em outros estudos realizados com profissionais de enfermagem^(44,45)^. Nesses profissionais, a CT tem sido apontada como um indicador fundamental para monitorar e possibilitar a intervenção precoce em aspectos de saúde do trabalhador, considerando a dimensão interna física e mental e aspectos externos relacionados a condições de trabalho, reverberando em melhorias na QV e impulsionando a produtividade geral da organização^(37,46)^.

A sonolência diurna é um aspecto que tem sido demonstrado com peculiar importância neste estudo, assim como em outros, envolvendo profissionais de enfermagem, pois os índices de sonolência diurna e sintomas de insônia têm sido alarmantes e mostram-se significativamente associados à QV. Melhores escores de QV, em todos os domínios, já foram anteriormente associados ao tempo médio de sono^(34,47,48)^. O desafio é adequar o sono ao ritmo de vida e de trabalho dos profissionais de enfermagem, considerando sua vulnerabilidade ao risco, fadiga frequente e necessidade de estar sempre atento e tomando decisões rápidas e sob pressão como consequência da característica do trabalho. Para além dos prejuízos na saúde ocupacional, a sonolência diurna excessiva está diretamente associada à exposição a riscos de erros e acidentes de trabalho, e isso significa aumento do risco de morbidade e mortalidade para si e para os usuários do serviço^(47,48)^.

No presente estudo, quase metade dos participantes considerava-se neutra em relação à satisfação no trabalho, abaixo do encontrado por outros autores^(26,32)^. Já o clima organizacional ruim, na maioria dos fatores, foi ao encontro de outros achados envolvendo profissionais de enfermagem^(26,49)^. A análise de regressão linear múltipla também demonstrou associação estatística significativa entre a satisfação com o trabalho e o componente mental da QV, enquanto o clima organizacional, nos fatores apoio da chefia e da organização e controle/pressão, impactou de forma significativa o componente físico.

Estudos têm buscado compreender de que maneira a percepção dos trabalhadores de enfermagem sobre seus contextos de trabalho e seu comportamento nas organizações levam em conta a carga positiva e negativa do ambiente laboral e suas influências na satisfação, motivação, bem-estar e qualidade do serviço prestado. Essa análise é particularmente importante na enfermagem, para que seja possível entender como o trabalhador é afetado pelas condições de trabalho, o relacionamento com colegas, empresa e chefias, a comunicação interpessoal, os benefícios e recompensas, bem como relacionar estatisticamente as dimensões do clima organizacional com a satisfação no trabalho, motivação para trabalhar, intenção de rotatividade profissional, entre outros^(26,32,37,42,49)^.

Estudo realizado com enfermeiros no Irã, antes da pandemia, relatou melhora da QV quando houve aumento do apoio e suporte dos superiores da instituição, redução da pressão no ambiente de trabalho, desenvolvimento de programas que melhoraram a felicidade e vitalidade e que foram capazes de desenvolver, na comunidade, uma mentalidade positiva em relação à profissão e compreensão dos problemas físicos e mentais enfrentados pelos trabalhadores^(32)^. Na mesma época, estudo canadense, realizado com profissionais de enfermagem, mostrou que melhorias no ambiente de trabalho e *status* no emprego melhoraram a assistência prestada aos pacientes e a QV relacionada à saúde dos profissionais. Além disso, melhora no ambiente de trabalho, melhores recursos humanos e materiais e carga de trabalho menor relacionaram-se positivamente à QV dos avaliados. No referido estudo, sugeriu-se que é mais significativo focar em melhora no ambiente de trabalho do que em fatores individuais. Embora a resiliência pessoal seja um preditor de QV, os fatores do trabalho, como adequação de recursos, impactaram mais^(21)^.

Melhorias na QV das pessoas podem ser acessadas ao investir no aumento da satisfação profissional, motivação no trabalho, liberdade de expressão dentro do serviço, orgulho da profissão, melhores condições de trabalho, benefícios oferecidos pela empresa, igualdade no tratamento dos funcionários, treinamentos e ambiente de aprendizagem, nível de participação nas decisões, nível de responsabilidade, relacionamento interpessoal, comunicação aberta e honesta, clareza de papéis, redução da pressão sobre o trabalhador, minimização do estresse e investimento no aumento da CT^(31,37)^.

### Limitações do estudo

Este estudo apresenta limitações relacionadas ao delineamento transversal e à amostra, que se restringiu aos profissionais de enfermagem, que são agentes públicos de instituição única.

### Contribuições para a área da enfermagem

Identificar e abordar esses fatores apresenta implicações para todos os profissionais de enfermagem e para a instituição, uma vez que ter a consciência da própria QV é condição sem a qual não há mobilização para buscar melhorias. Aos líderes, cabe reconhecer as necessidades de sua equipe e buscar a promoção da harmonia e equilíbrio das relações no trabalho. A instituição precisa reconhecer a importância da QV dos profissionais que a compõem, para que possa ofertar melhor ambiente laboral, com políticas e recursos adequados, além de poder organizar os fluxos de trabalho, para que os indivíduos desenvolvam e apliquem suas competências pessoais e profissionais em benefício da população por eles assistidos.

## CONCLUSÕES

A QV foi considerada baixa para os profissionais de enfermagem na amostra analisada. O componente físico estava inversamente associado à renda familiar. O clima organizacional no fator “controle/pressão” também afetou a QV física. Todavia, a realização de atividade física e um melhor apoio da chefia e da organização resultaram em melhorias nesse quesito. Já o componente mental foi prejudicado pela baixa satisfação no trabalho e autoavaliação da saúde física ruim. Por outro lado, foi mais bem avaliado por profissionais mais jovens. Ambos os componentes da QV pioraram quando os profissionais de enfermagem referiram doenças relacionadas ao trabalho, baixa CT e agravamento da sonolência diurna.
